# Testosterone regulates Arp2/3 expression by DNA methylation in hippocampus

**DOI:** 10.1186/1750-1326-7-S1-S27

**Published:** 2012-02-07

**Authors:** Liang Zhou, Ximeng Zhang, Xuefei Wu, Huan Yang, Kaiyin Zhong, Hecheng Wang, Ting Zhou, Tianxiao Sheng, Yawei Tong, Dongsheng Fan, Dehua Chui

**Affiliations:** 1 Neuroscience Research Institute, Key Laboratory for Neuroscience, Ministry of Education & Ministry of Public Health, Health Science Center, Peking University, Beijing 100191, China

## Background

The Arp2/3 complex is a seven-protein assembly that is critical for actin nucleation and branching in cells. Although some DNA methylation patterns are altered by steroid hormone exposure in the developing brain, less is known about how changes in steroid hormone levels influence Arp2/3 complex DNA methylation patterns in the adult brain. Steroid hormones act in the adult brain to regulate gene expression. Specifically, the expression of the Arp2 within adult brain is dependent upon testosterone exposure.

## Method and results

Castration dramatically reduces and testosterone replacement restores Arp2 expression in hippocampus. As decreases in mRNA expression are associated with increases in DNA promoter methylation, we explored the hypothesis that Arp2 expression in the adult brain is maintained through sustained epigenetic modifications of the Arp2 gene promoter. We find that castration of adult male rats resulted in decreased Arp2 mRNA expression and increased methylation of specific CpG sites within the Arp2 promoter in the hippocampus. Similarly, castration significantly increased estrogen receptor α (ERα)/androgen receptor(AR) mRNA expression and decreased ERα promoter methylation within the hippocampus. These changes were prevented by testosterone replacement.

## Conclusion

This suggests that the Arp2 DNA promoter methylation status of some steroid responsive genes in the adult brain is actively maintained by the presence of circulating steroid hormones. The maintenance of methylated states of some genes in the adult brain by the presence of steroid hormones may play a role in the cytoskeleton systems.

